# Abnormally Enlarged Singular Thebesian Vein in Right Atrium

**DOI:** 10.7759/cureus.16300

**Published:** 2021-07-10

**Authors:** Dilip Kumar, Amit Malviya, Bishwajeet Saikia, Bhupen Barman, Anunay Gupta

**Affiliations:** 1 Cardiology, Medica Institute of Cardiac Sciences, Kolkata, IND; 2 Cardiology, North Eastern Indira Gandhi Regional Institute of Health and Medical Sciences, Shillong, IND; 3 Anatomy, North Eastern Indira Gandhi Regional Institute of Health and Medical Sciences, Shillong, IND; 4 Internal Medicine, North Eastern Indira Gandhi Regional Institute of Health and Medical Sciences, Shillong, IND; 5 Cardiology, Vardhman Mahavir Medical College (VMMC) and Safdarjung Hospital, New Delhi, IND

**Keywords:** thebesian veins, enlargement, venous drainage, right atrium, anatomy

## Abstract

Thebesian veins in the heart are subendocardial venoluminal channels and are usually less than 0.5 mm in diameter. The system of TV either opens a venous (venoluminal) or an arterial (arterioluminal) channel directly into the lumen of the cardiac chambers or via some intervening spaces (venosinusoidal/ arteriosinusoidal) termed as sinusoids. Enlarged thebesian veins are reported in patients with congenital heart disease and usually, multiple veins are enlarged. Very few reports of such abnormal enlargement are there in the absence of congenital heart disease, but in all such cases, they are multiple and in association with coronary artery microfistule. We report a very rare case of a singular thebesian vein in the right atrium, which was abnormally enlarged. It is important to recognize because it can be confused with other cardiac structures like coronary sinus during diagnostic or therapeutic catheterization and can lead to cardiac injury and complications if it is attempted to cannulate it or pass the guidewires.

## Introduction

The thebesain veins (TVs) or the smallest cardiac venous system essentially consists of thin vessels, lacunae, or channels, which are subendocardial in distribution and act as communication between intramyocardial vessels with the cardiac chambers [1]. In doing so, the system of TV either opens a venous (venoluminal) or an arterial (arterioluminal) channel directly into the lumen of the cardiac chambers or via some intervening spaces (venosinusoidal/arteriosinusoidal) termed as sinusoids. These sinusoids are a highly anastomosing network of thin-walled irregular channels present within the myocardial musculature, which are further continuous with some inter-trabecular spaces. The inter-trabecular space, in turn, empties directly into the chambers of the heart. Therefore, collectively the system of TV consists of the following patterns: venoluminal, arterioluminal, venosinusoidal, and arteriosinusoidal . Out of all, the venoluminal is the most common pattern. The valveless opening of the TV is more commonly seen in the right atrium and right ventricle and is usually less than 0.5 mm in diameter [2]. In the right atrium, TVs are found to be numerous and are densely packed. They classically drain the walls of the right auricle especially near the sinoatrial node and directly open into the right auricle [1].

Even though the volume of blood shunting across the TV is not very significant (0.12% and 0.43% of the total flow), they are regarded as important channels for providing nourishment and drainage to the myocardium [3,4]. There are many theories postulated, which suggest the different origins of thebesian vessels from the rest of the coronary venous system. TVs are the embryological representation of inter-trabecular circulation, which were independent of the main venous system [1,5]. At a later stage, the secondary growth of this inter-trabecular system toward the epicardium establishes communication of the former with the rest of the great cardiac venous system [5].

## Case presentation

**Figure 1:**
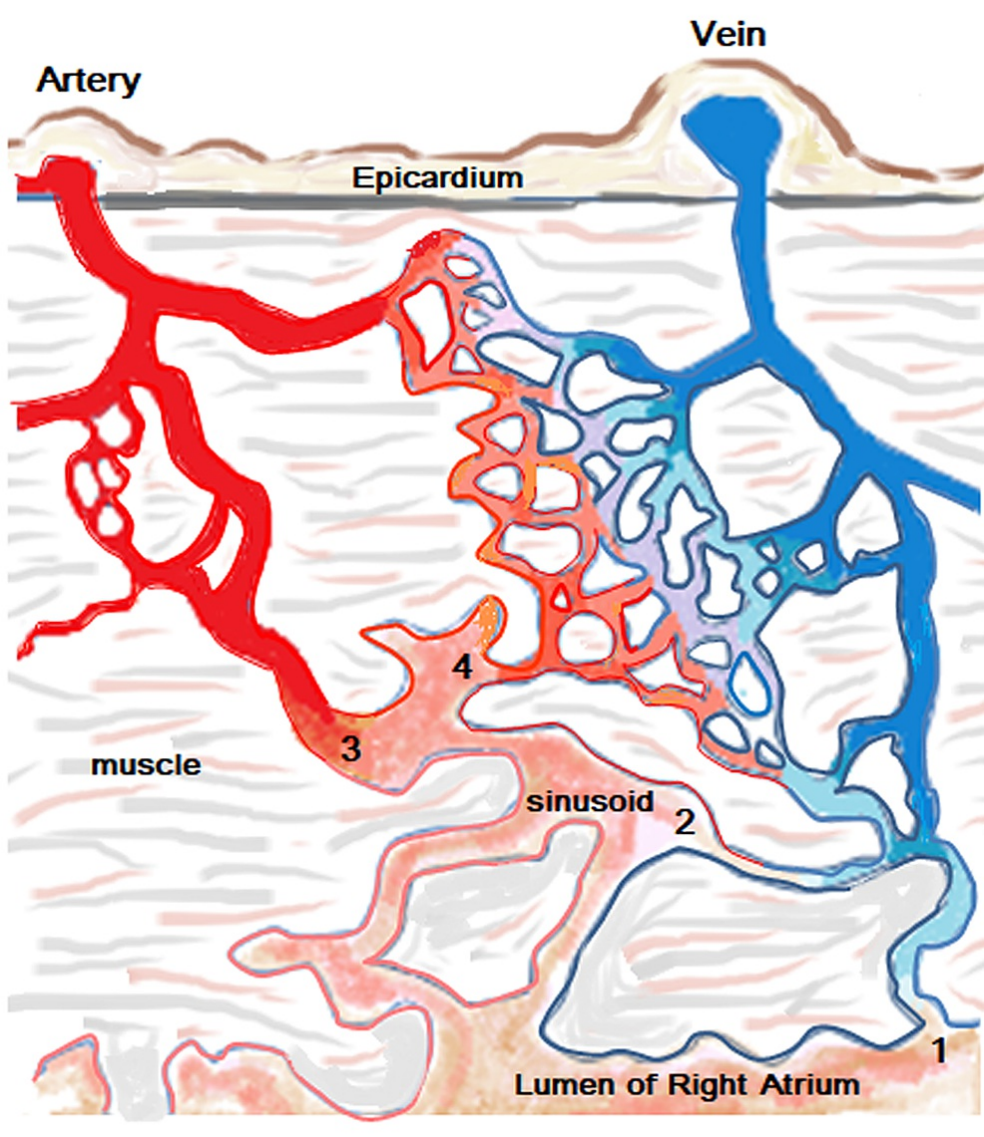
Schematic representation showing patterns of thebesian vessels: 1: venoluminal vessels that connect intramural venules with the lumen of the cardiac chambers, 2: venosinusoidal vessels that connect intramural venules with subendocardial sinusoids and then the lumen, 3: arterioluminal vessels that connect arterioles with the atria/ventricles without traversing the capillary beds, 4: arteriosinusoidal vessels that connect arterioles with the sinusoidal spaces. (The figure was created by Dr. Bishwajeet Sakia.)

A 75-year-old gentleman with a previous history of coronary artery bypass surgery (12 years back) presented with progressive dyspnoea of New York Heart Association (NYHA) class III. Upon evaluation, he was found to have severe left ventricular dysfunction with an ejection fraction of 30% and a left bundle branch block pattern on ECG with a QRS duration of 152 milliseconds. Coronary angiography revealed native double vessel disease with patent grafts to left anterior descending and posterior descending arteries. There was no evidence of coronary artery fistula in the levo phase. After the optimization of medical therapy and patient consent, he was taken up for cardiac resynchronization therapy (CRT) device implantation. While attempting to cannulate the ostium of the coronary sinus, an abnormally enlarged Thebesian vein, was serendipitously cannulated. This abnormal and singularly enlarged vein was venoluminal type of thebesian vein (Figure 1). 

**Figure 2:**
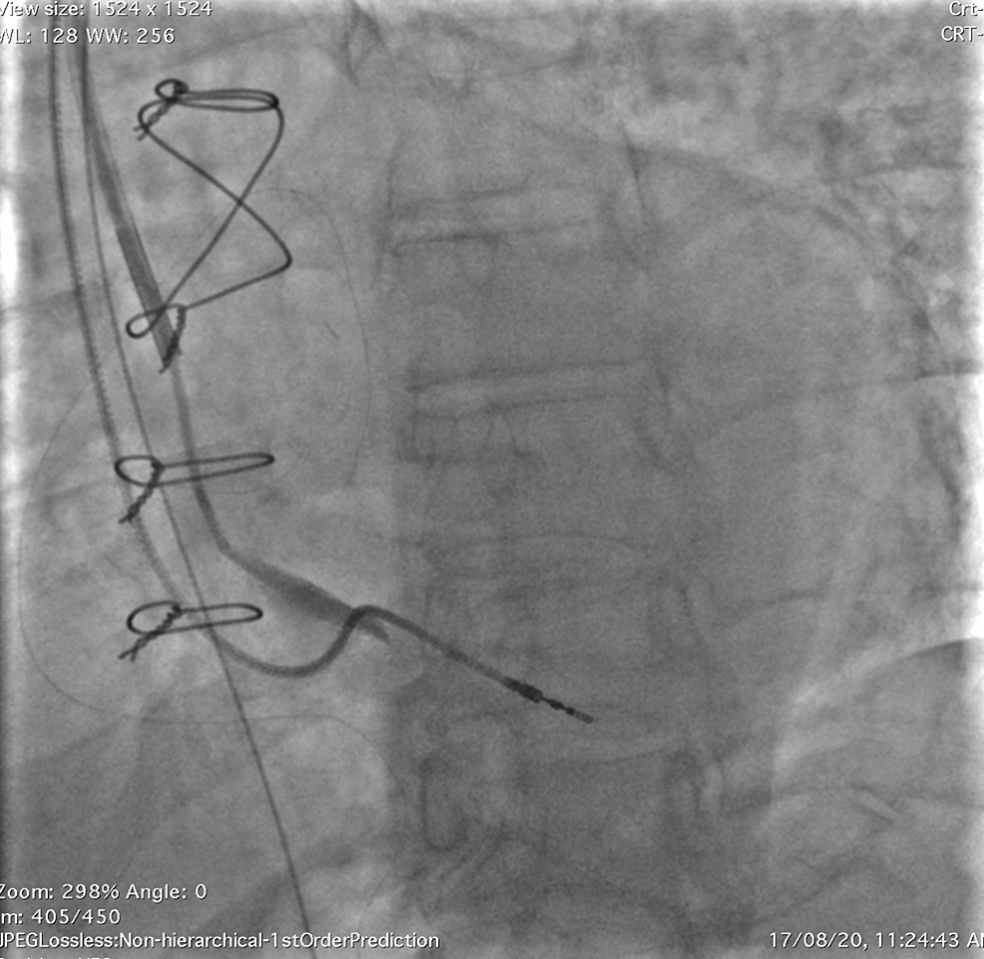
The still image depicts the serendipitously cannulated, singularly enlarged thebesian vein draining the wall of the right atrium in the left anterior oblique view.

**Figure 3:**
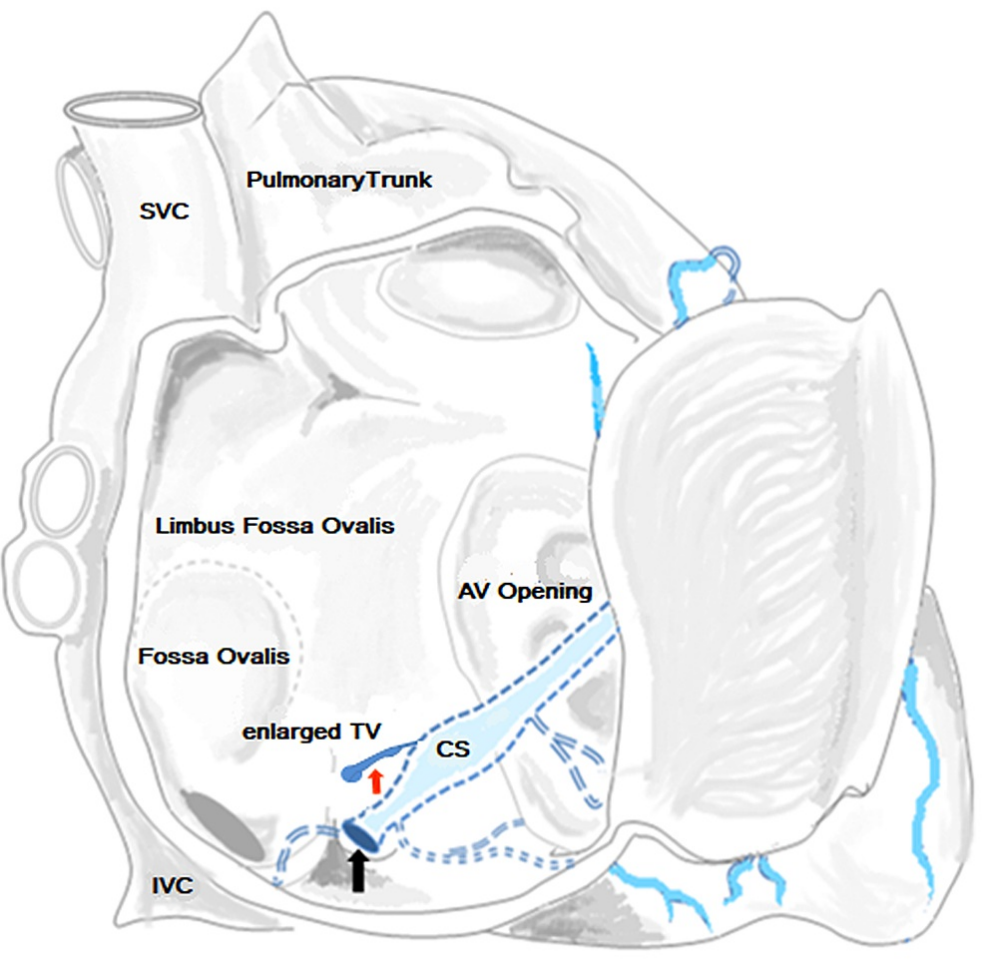
Schematic representation of the interior of the right atrium (dissected) showing an abnormally enlarged thebesian vessel (red arrow) opening superoanteriorly to the ostium of the coronary sinus (black arrow). TV = Thebesian Vein, CS = Coronary sinus, SVC = Superior vena cava, IVC: Inferior vena cava. (This image was created by Dr. Bishwajeet Saikia.)

Upon injection of contrast, the thebesian was promptly visualized in the right atrium (Figures 2, 3, Video 1).

**Video 1:** The serendipitously cannulated, singularly enlarged thebesian vein draining the wall of the right atrium in the left anterior oblique view.

The ostium of this singularly enlarged TV was 1.3 mm in diameter and the maximum diameter was measured to be 4.49 mm. The ostium of this enlarged TV was located 4 mm superoanteriorly to the ostium of the coronary sinus. The large TV opening, which was connecting the endocardial surface of the right atrium and the coronary sinus, was initially mistaken to be the ostium of the coronary sinus itself. But subsequently, we could cannulate the true coronary sinus ostium and the implantation was completed successfully (Video 2).

**Video 2:** The complete implant of cardiac resynchronization therapy device.

The patient improved following the procedure and was discharged with improved symptoms and was stable at follow-up in the outpatient clinic.

## Discussion

First described by Vieussens in the year 1706, the TV represents the primitive vascular network supplying the endocardium during embryogenesis [6]. They serve to drain the myocardium of all the cardiac chambers but are specifically more abundant in the right atrium [1,7,8]. Thus, these vessels provide an alternative route for cardiac nourishment, which can account for up to 30% of the cardiac venous drainage [1]. Additionally, TVs are of significance for providing vascular drainage by contributing towards the right to left shunting of deoxygenated blood. Although not proven they are also suggested to be able to maintain the myocardial blood supply in episodes of coronary arterial occlusion [7]. The TV has been known to drain the atrioventricular node after which they commonly open into the right atrium, 1 cm anteriorly or posteriorly to the ostium of the coronary sinus [9]. The ostium of the abnormally enlarged TV in our reported case opened 4 mm superoanteriorly to the ostium of the coronary sinus.

The TV has shown to be of clinical significance only in a few selected clinical scenarios or anecdotal case reports. An enlarged TV may be of clinical relevance during cardiac surgery, causing a large amount of retrograde cardioplegia solution to be shunted into the ventricles without reaching the capillary beds, making cardioplegia much less efficient [10]. There are reports of cases where persistent TV was causing secondary acute or chronic myocardial ischemia [11,12]. It has been shown that the TVs have an increased diameter in congenital heart disease (CHD) with abnormal drainage of the coronary sinus and could be large enough to provide alternative access to the coronary venous system and the ventricles [13]. Moreover, in patients with CHD angiographically detectable Thebesian veins may represent global changes in the coronary circulation, their dilation is a dynamic process capable of both progression and regression and can be graded from grade 0 to grade 3. However, in most cases, TV is of no clinical significance [14].

We report a very rare case of singularly and abnormally enlarged TV of venoluminal pattern, draining the wall of the right atrium, in the absence of CHD. Such findings may be of clinical significance in patients who are undergoing cardiac catheterization or intervention and can be confused with coronary sinus. Any attempts to cannulate such enlarged TV may lead to serious cardiac injury.

## Conclusions

The TVs are the smallest cardiac venous system. The system of TV consists of the following patterns: venoluminal, arterioluminal, venosinusoidal, and arteriosinusoidal, and the venoluminal TV is the most common pattern. TV assumes clinical significance only in a few selected clinical scenarios like CHD or a rare cause of myocardial ischemia. We report a very rare case of singularly and abnormally enlarged TV of venoluminal pattern, draining the wall of the right atrium, in the absence of CHD. Such findings may be of clinical significance in patients who are undergoing cardiac catheterization or intervention and can be confused with coronary sinus. Any attempts to cannulate such enlarged TV could lead to cardiac injury.
